# Tumor-infiltrating immune cells in hepatocellular carcinoma: Tregs is correlated with poor overall survival

**DOI:** 10.1371/journal.pone.0231003

**Published:** 2020-04-02

**Authors:** SiZhe Yu, Yu Wang, Jia Hou, WenYuan Li, Xiao Wang, LuoChengLing Xiang, DeLi Tan, WenJuan Wang, LiLi Jiang, Francois X. Claret, Min Jiao, Hui Guo

**Affiliations:** 1 Department of Medical Oncology, The First Affiliated Hospital of Xi'an Jiaotong University, Xi’an, Shaanxi, PR China; 2 Department of Respirology, The Second Affiliated Hospital of Xi'an Jiaotong University, Xi’an, Shaanxi, PR China; 3 Department of Systems Biology, The University of Texas MD Anderson Cancer Center, Houston, TX, United States of America; 4 Key Laboratory of Environment and Genes Related to Diseases, Xi'an Jiaotong University, Ministry of Education of China, Xi’an, Shaanxi, PR China; University of Calgary, CANADA

## Abstract

Systematic interrogation of tumor-infiltrating immune cells (TIICs) is key to the prediction of clinical outcome and development of immunotherapies. However, little is known about the TIICs of hepatocellular carcinoma (HCC) and its impact on the prognosis of patients and potential for immunotherapy. We applied CIBERSORT of 1090 tumors to infer the infiltration of 22 subsets of TIICs using gene expression data. Unsupervised clustering analysis by 22 TIICs revealed 4 clusters of tumors, mainly defined by macrophages and T cells, with distinct prognosis and associations with immune checkpoint molecules, including PD-1, CD274, CTLA-4, LAG-3 and IFNG. We found tumors with decreased number of M1 macrophages or increased regulatory T cells were associated with poor prognosis. Based on the multivariate Cox analysis, a nomogram was also established for clinical application. In conclusion, composition of the TIICs in HCC was quite different, which is an important determinant of prognosis with great potential to identify candidates for immunotherapy.

## Introduction

As the most common form of primary liver cancer, hepatocellular carcinoma (HCC) is one of the leading causes of cancer-related deaths worldwide [1]. Most patients are diagnosed at an advanced stage so that they cannot benefit from radical resection, and are refractory to targeted drugs and chemotherapy [2, 3]. The approval of nivolumab and pembrolizumab by the U.S. Food and Drug Administration for the treatment of patients with HCC is a strong hint that immunotherapy will introduce a new era of HCC therapy [4–6]. However, immunotherapy only leads to 10–20% clinical responses. Several factors such as expression of programmed cell death-Ligand 1 (PD-L1), tumor mutational loads (TMB), and tumor-infiltration immune cells (TIICs) provide certain correlation with patient responses for immunotherapy[7]. Therefore, identification of patients’ immune status is becoming much more critical so that the specific population of patients could benefit from immunotherapy.

The HCC immune feature, due to the chronic inflammation and cirrhosis in most HCC patients, is complicated and varies dynamically[8, 9]. The heterogeneity of the HCC ecosystem influences the growth, invasion, and metastasis of tumor and efficacy of treatment[4]. By far, some studies have unveiled the prognostic significance of some TIICs and immune molecules, such as tumor associated macrophages (TAMs), dendritic cells (DCs), natural killer (NK) cells, PD-L1, PD-L2, and TIM-3 in HCC[10–14]. However, most investigations focused on only one or two cell types by immunohistochemistry-based analysis. The immune features, however, is composed by a number of cells and factors with complex interactions[8, 15–17]. Thus, there might be a lot of inconsistent results in different studies. A comprehensive study on the distinct immune landscapes rather than a single marker and the impact on prognosis of HCC patients and subsequently implications on disease management remains unexplored. Integrating genomic profiles overcome the shortcoming of IHC-based researches[18–20]. Simultaneously, bioinformatics have made the dream of studying the correlation between immune infiltrations in large scale of public profiles with clinical outcome come true.

Hence, we applied CIBERSORT[21], estimating the relative infiltration of 22 subsets of TIICs, to 1090 cases of HCC. Such multidimensional analysis have helped us gain a deep understanding of the immune landscape of HCC, identified the relationship between different TIICs infiltration patterns and immune checkpoint molecules, and its impact on overall survival (OS), providing evidence for immunotherapy and prediction of survival in HCC patients.

## Materials and methods

### Gene expression data

**Fig 1:**
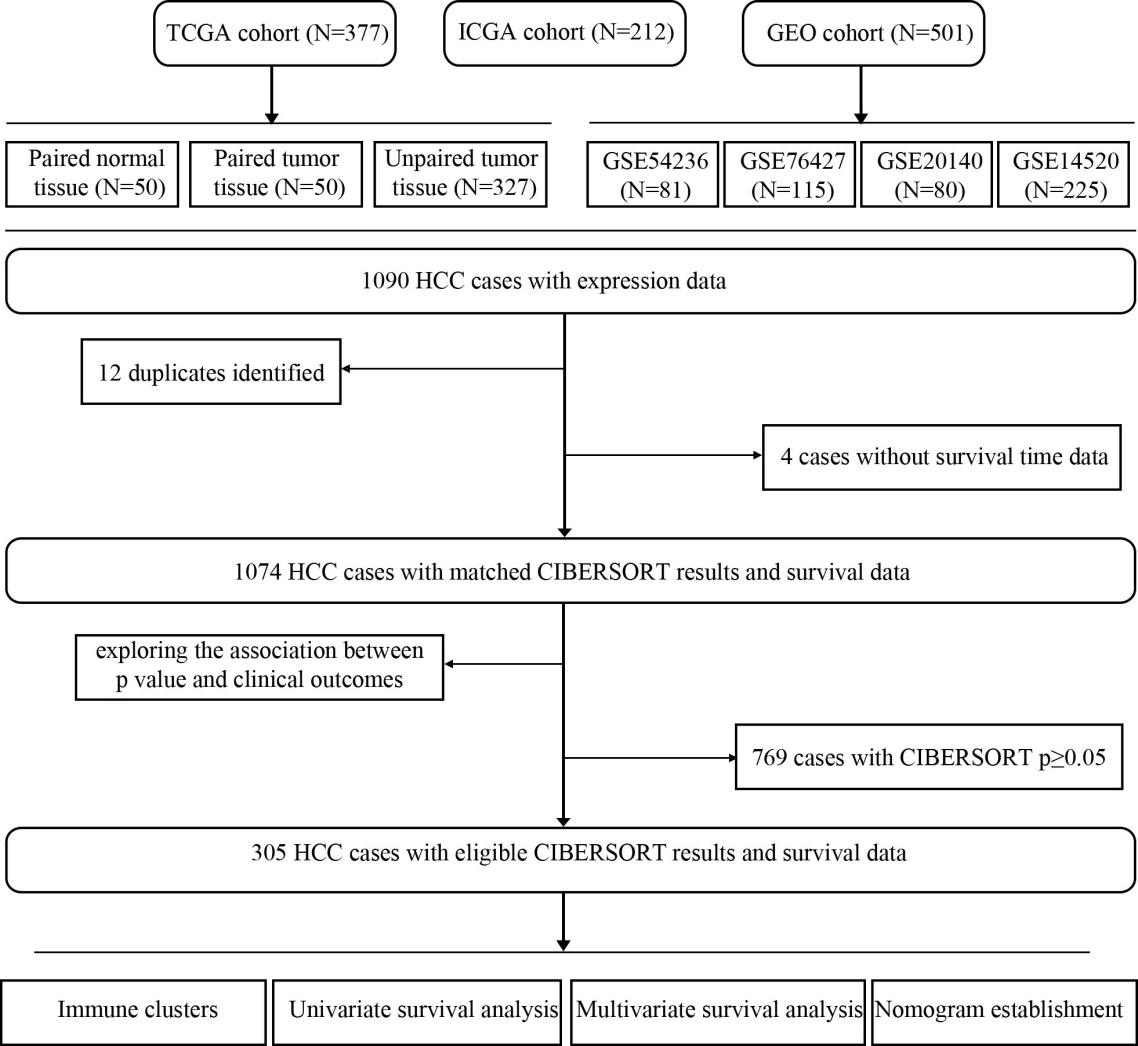
Study flowchart of design and number of samples (N).

This study used public data. Gene expression datasets with corresponding clinical information of HCC were downloaded from The Cancer Genome Atlas (TCGA), International Cancer Genome Consortium (ICGC) and Gene Expression Omnibus (GEO) (up to March 28, 2019, the related links of these datasets were provided). The gene expression profile matrix files from GSE54236, GSE76427, GSE20140 and GSE14520 were downloaded from the GEO database. Cases identified as replicates were removed. In total, 1090 cases were available for analysis. Details of the samples included were shown in Fig 1 as a flowchart. RNA sequencing data were firstly normalized using voom [22], converting different values more similar.

### Analysis of tumor infiltrating immune cells

Transcriptome data collected were first normalized using voom and run the CIBERSORT (http://cibersort.stanford.edu/) at 1,000 permutations to evaluate the relative proportions of 22 types of tumor infiltrating immune cells (TIICs)[21]. Immune cytolytic activity by Rooney et al. [23], counting the geometric mean of two immune markrs, GZMA and PRF1, was calculated in TCGA dataset for the comparison of the two methods. Expression levels of immune checkpoint molecules in the TCGA, ICGC, and GEO cohorts were normalized with mean value = 0 and standard deviation = 1. Tumor mutation burden for the TCGA dataset was downloaded from TCGA and calculated using maftool (http://www.r-project.org/).

### Statistical analysis

Associations between proportions of 22 TIICs and overall survival (OS) were tested using Cox regression with samples with CIBERSORT p value < 0.05. To investigate whether there were different clusters of immune infiltration associated with prognosis, we conducted hierarchical clustering. We combined the Elbow, Silhouette and used Ward’s method to explore the optimal k number of clusters. Correlations between different subsets of distinct immune cells were analyzed using Pearson correlation. Univariate and multivariate Cox regression analysis was performed to evaluate the prognostic value of 22 TIICs. The log-rank test was performed to assess the OS between 2 groups based on median in Kaplan-Meier plots. A nomogram was established based on the multivariate Cox regression analyses using rms package (https://cran.r-project.org/web/packages/rms/index.html). The performance of the nomogram was assessed using Harrel’s concordance index (C-index) and comparing the predicted and actual probabilities for OS. All analysis was conducted using R version 3.5.2 or SPSS Statistics version 24.0.

## Results

### High immune infiltration improved the outcome of HCC patients

**Fig 2:**
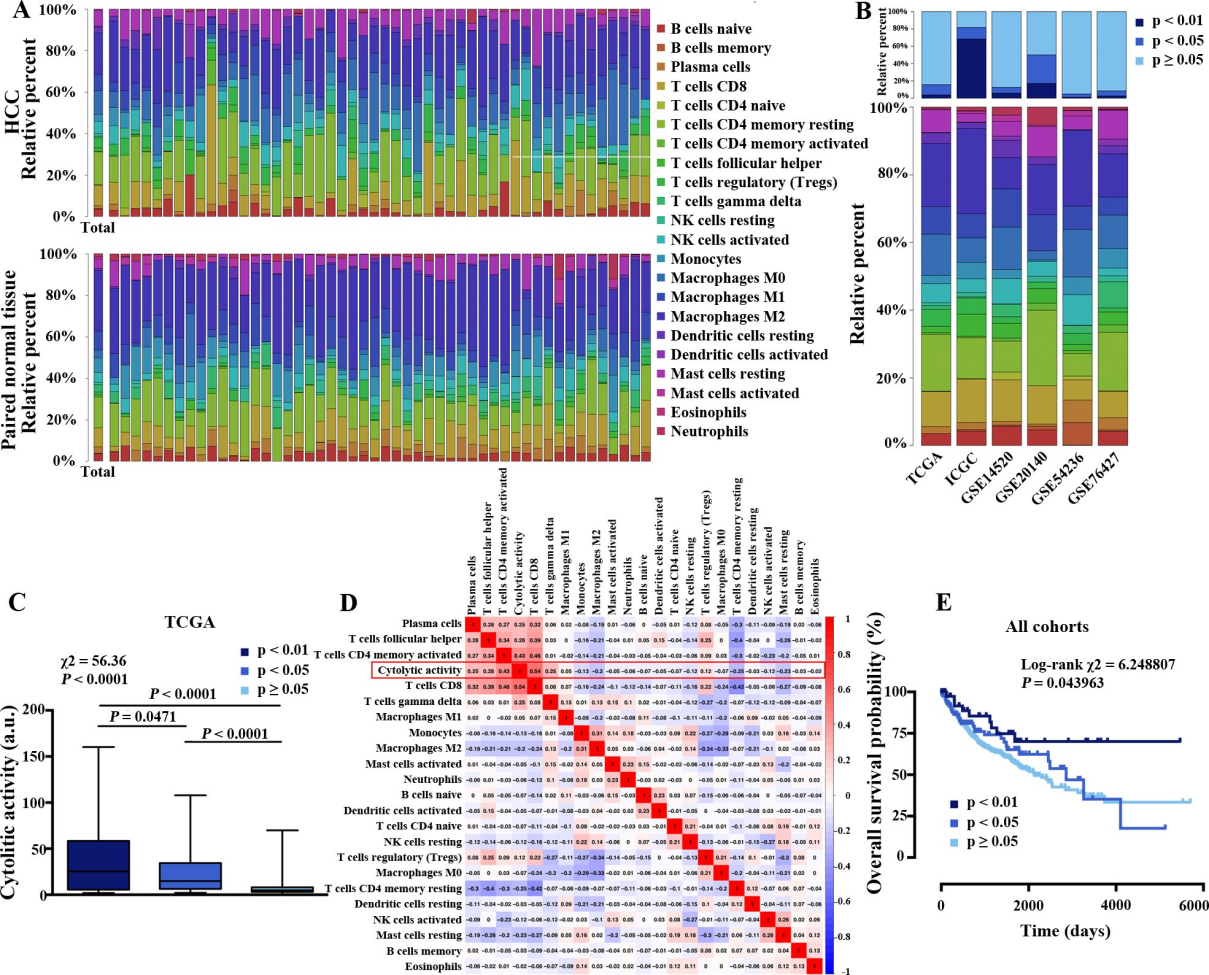
The landscape of immune infiltration in HCC. (A) The different immune infiltration of 22 tumor-infiltrating immune cells (TIICs) between HCC and paired normal tissue; (B) The difference of TIICs proportions and CIBERSORT p-value; (C) The association between immune cytolytic activity and CIBERSORT p-value in the TCGA cohort, a.u., arbitrary units; (D) Peason’s correlation matrix of 22 TIICs and immune cytolytic activity in the TCGA cohort; (E) Kaplan-Meier survival analysis of groups with different CIBERSORT p-value.

CIBERSORT evaluated the relative proportions of 22 subsets of tumor-infiltrating immune cells (TIICs) with distinct functions. Using CIBERSORT, we first compared the different infiltrations between HCC tissues and paired normal tissue, as shown in Fig 2A and S1 Fig. Fig 2B summarizes immune infiltration of the included studies, including gene expression data from The Cancer Genome Atlas (TCGA), International Cancer Genome Consortium (ICGC) and Gene Expression Omnibus (GEO), totalling 1090 cases of HCC. The proportions of immune cells in HCC vary significantly between both intra-and intergroup.

The CIBERSORT p value varied among studies. The CIBERSORT p value reflects the proportion of a sample that comprises immune cells versus non-immune cells[21]. Cytolytic activity, another *in silico* parameter of immune infiltration, was defined as the geometric mean of GZMA and PRF1 expression [23]. We tested cytolytic activity with samples with different p value in TCGA dataset (Fig 2C) and found a strong ordinal relationship. Cytolytic activity was most strongly correlated with the proportion of CD8+ T Cells (Pearson correlation = 0.54) and CD4+ memory activated T cells (Pearson correlation = 0.43) in the TCGA cohort of HCC at a CIBERSORT p < 0.05 (Fig 2D). The relationship between cytolytic activity and CIBERSORT p value strongly suggests that the smaller p value reflects the higher immune infiltration.

We next explored the association between p value and overall survival (OS). p < 0.01, which means a higher infiltration of immune cells, was associated with improved overall survival (Fig 2E). However, there are no significantly differences between p≥0.01 and <0.05 and p≥0.05 groups. In conclusion, these results revealed that high immune infiltration might improve the outcome of HCC patients.

### Immune clusters were associated with outcome

**Fig 3:**
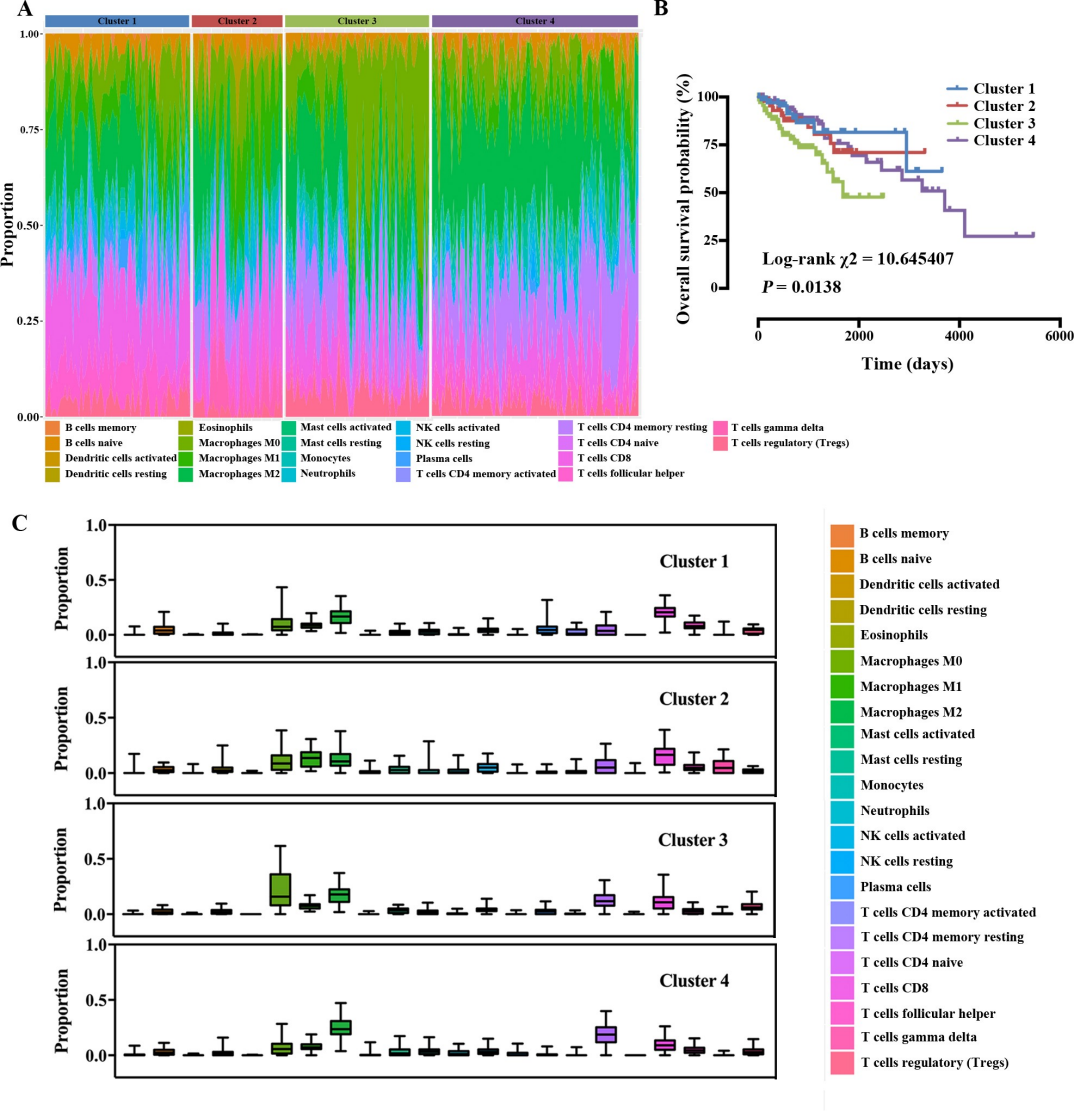
Immune clusters associated with immune infiltration and outcome. (A) Hierarchical clustering based on 22 TIICs proportions; (B) Kaplan-Meier survival plots of patients within different clusters; (C) Box plots summarising immune cell subset proportions by cluster.

As the fact that the infiltration of TIICs partly reflects the outcome of HCC patients, we next investigated whether different patterns of immune infiltration based on the 22 TIICs has varied outcome. The optimal number of clusters was determined by combining Elbow and Silhouette method, appeared to be for k = 4 (S2 Fig). Cell proportions by cluster depicting the four clusters cross all sample from TCGA, ICGC and GEO are shown in Fig 3A. Clusters were associated with OS (Fig 3B). Distributions of TIICs of each cluster as box plots are shown in Fig 3C and S3 Fig. As shown in Fig 3C, the distribution of macrophage and T cells varies greatly among clusters. Cluster 3, defined by a high level of macrophage with a significantly low level of M1 macrophages and a low level of T cells with a high level of Tregs, was associated with poor outcome. Cluster 1 and Cluster 2, defined by a high level of T cells, especially CD8+ T cells, and low level of macrophages, were associated with better outcome. However, Cluster 4, with complex cell proportions, had a complex outcome.

### Immune clusters were associated with immune checkpoint molecules

**Fig 4:**
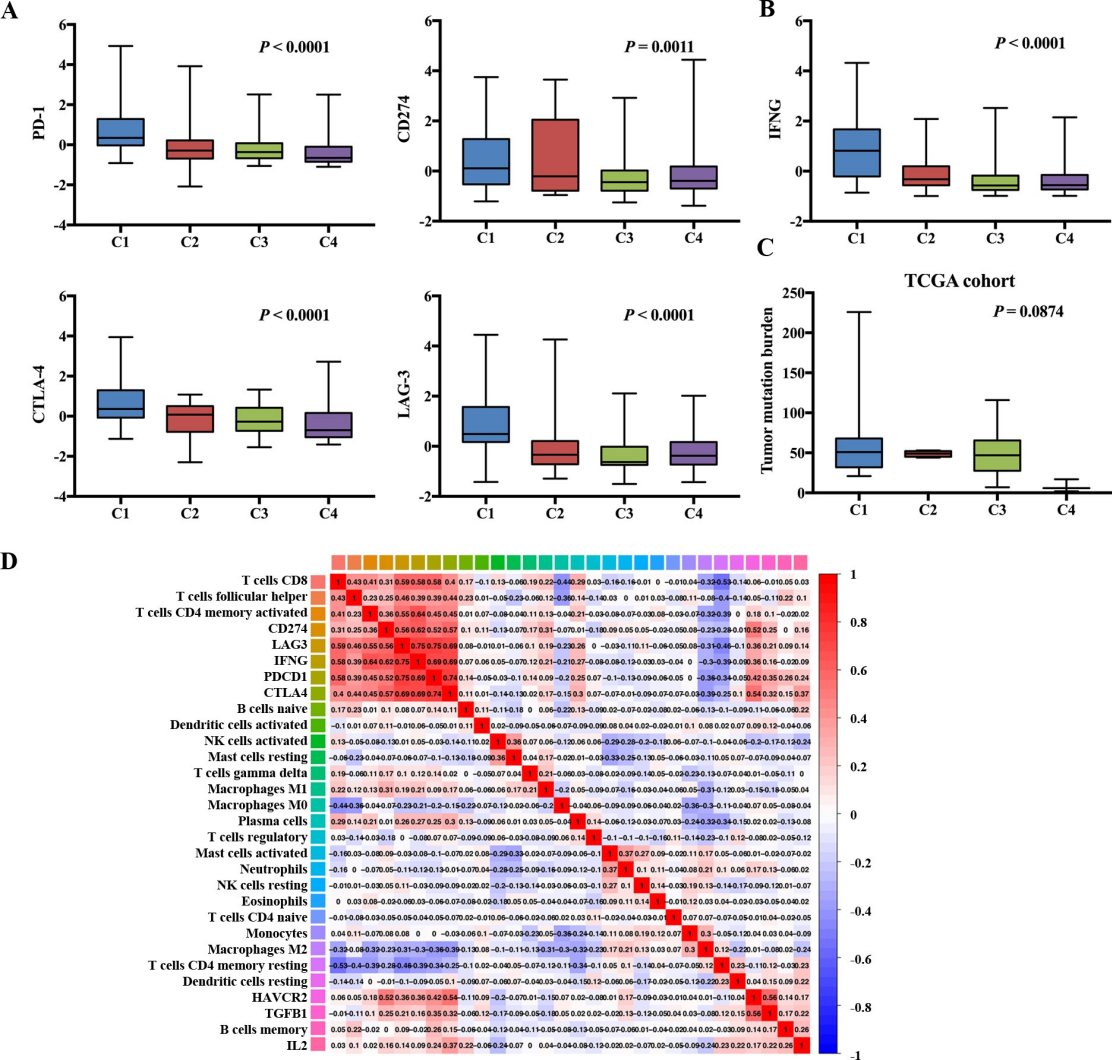
Immune clusters associated with immune checkpoint molecules. (A) Evaluation of the immune checkpoint molecules expression in different immune clusters; (B) IFNG expression in different immune clusters; (C) Evaluation of the TMB in different immune clusters in the TCGA cohort; (D) Correlation matrix of all 22 TIICs and immune checkpoint molecules expression.

To explore the associations between infiltration of TIICs and immune checkpoint molecules, we tested the expression of some important immune molecules, which have been verified to be associated with the efficacy of immune checkpoint inhibitors[24–26], among clusters. As the expression profiles of each cohort were obtained using different technologies, we first normalized of the expression levels of immune checkpoint molecules in the TCGA, ICGC, and GEO cohorts with mean value = 0 and standard deviation (SD) = 1. Expressions of PD-1, CD274, CTLA-4, LAG-3 and IFNG were significantly higher in Cluster 1, and moderate in Cluster 2, lower in Cluster 3 and Cluster 4 (Fig 4A and 4B and S4 Fig). The tumor mutational burden (TMB) has been shown to correlate with patient response to both CTLA-4 and PD-1 inhibition in several tumor types [27–29]. In the TCGA cohort, TMB was not significantly different but had the same trend among different clusters (Fig 4C). Collectively, the findings above suggest that the different clusters, which are determined by the different immune infiltration and molecular characteristics of tumors, influences clinical outcome. Also, the variability may be one of the factors influencing the efficacy of immunotherapy.

As shown in Fig 4D, CD8+ T cells and CD4+ memory activated T cells were strongly correlated with immune checkpoint molecules. Macrophages, depending on their phenotype, had opposing effects. In conclusion, correlation between the immune checkpoint molecules and TIICs demonstrated an immunosuppressive and exhausted tumor microenvironment in HCC, providing evidence for immunotherapy.

### Prognostic subsets of immune cells

**Fig 5:**
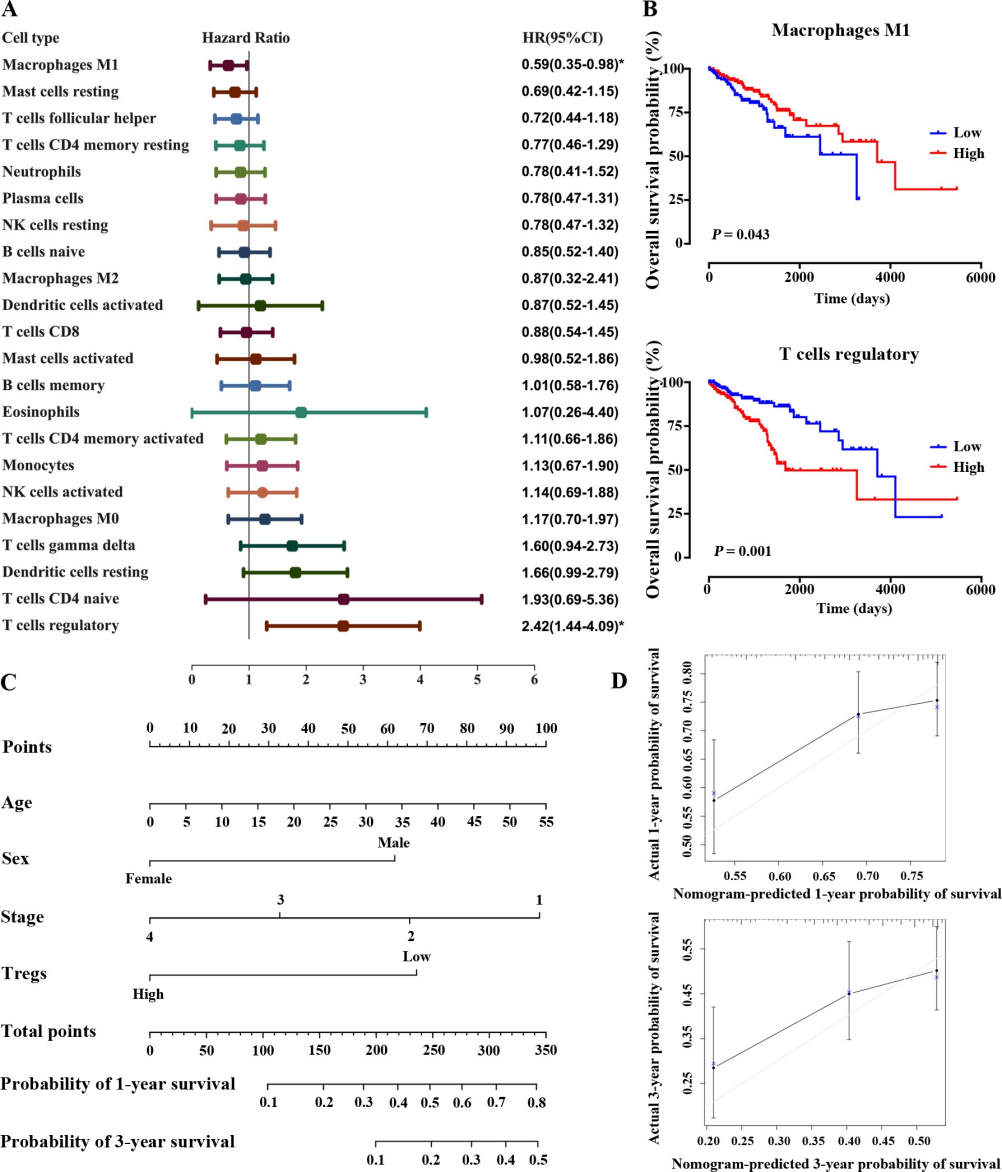
Prognostic subsets of TIICs in HCC. (A) Hazard Ratio (HR) and 95% confidence intervals (CI) limited to cases with CIBERSORT p value <0.05. *, P<0.05; (B) Kaplan-Meier survival analysis of patients within different proportion of TIICs by median; (C) Nomogram for predicting the probability of overall survival (OS) for HCC patients; (D) Calibration plots of the nomogram for predicting OS rate at 1 year and 3 years.

In the former part, we found immune clusters were associated with OS. To elucidate the specific cell types that are critical to survival, we investigated whether there were TIICs subpopulations correlated with HCC patients’ OS by univariate Cox regression analysis. The hazard ratio (HR) and 95% confidence intervals (CI) of the 22 TIICs were shown in Fig 5A. Among 22 immune cells, macrophage M1 (HR = 0.59, 95% CI = 0.35–0.98; *P* = 0.043) was significantly associated with improved OS, whereas Tregs (HR = 1.37, 95% CI = 1.08–1.73; *P* < 0.001) was significantly associated with poorer OS. Kaplan-Meier curve for the median proportions of TIICs associated with OS and the rest are shown in Fig 5B and S5 Fig. We also investigated whether immune checkpoint molecules statistically correlated with HCC patients’ overall survival, however, in this study, none of the markers was correlated with OS by univariate Cox regression analysis (S1 Table).

We next assessed whether those selected TIICs subsets are independent indicators in HCC patients. Multivariable analysis revealed that, besides age, sex and TNM stage, relative proportions of Tregs was an independent prognostic factor for OS, which might add prognostic value in clinical practice (Table 1).

**Table 1:** Multivariate Cox regression survival analysis in HCC patients.

Variable	Categories	Hazard ratio	95% CI	*P*-value
**Tregs**^**a**^	low,high	3.355	1.842–6.114	**0.000077**
**Macrophages M1**^**a**^	low,high	0.785	0.462–1.331	0.369
**Sex**	female/male	0.505	0.29–0.879	**0.016**
**Age**	<60/≧60years	0.435	0.248–0.764	**0.004**
**TNM stage**	I,II,III,IV	1.547	1.131–2.118	**0.006**

^a^ Selected immune cell subsets are those significantly associated with outcome in univariate Cox regression analysis as shown in Fig 5A.

To link our findings with clinical practice and provide a quantitative tool to predict the OS of HCC patients based on the multivariate Cox analysis as shown in Table 1, a nomogram was constructed (Fig 5C). The probability of 1 and 3 years overall survival for HCC patients was determined by the total points. Calibration is assessed by reviewing the plot of predicted probabilities from the nomogram versus the actual probabilities. The calibration plot with the CIs added is shown in Fig 5D. We also compared the accuracy of this nomogram (C-index: 0.689) with that composed of different parameters, such as nomogram determined by age (C-index: 0.527), sex (C-index: 0.575) and TNM stage (C-index: 0.607). In sum, these findings suggest that this nomogram was a good model for predicting overall survival in HCC patients.

## Discussion

In this study, using CIBERSORT, we firstly compared the infiltration of 22 TIICs between HCC and paired normal tissue, and among different cohorts, found significant change in both intra-and intergroup. Among the 22 TIICs subsets in HCC, macrophages was the most abundant, accounting for about 40%, in which M2, regarded as the tumor associated macrophage (TAM), make up 19%. It is worth noting that Tregs accounts for 4.84% in tumor tissue while only 1.38% in adjacent normal tissue with a 3.5 fold change increase (S1 Fig), may have important roles in pathogenesis of HCC, which is consistent with the previous research [30–32]. We next validated in HCC the CIBERSORT p value could reflect the immune infiltration. In further exploring the association between p value and overall survival, we found HCC patients with increased infiltration of immune cells have better outcome, emphasizing the significance of immune infiltration in HCC.

Next, we found that the proportions of immune cells in our clustering analysis had prognostic implications. Cluster 3, defined by a high level of macrophage with a significantly low level of M1 macrophages and a low level of T cells with a high level of Tregs, was associated with poor outcome. Different clusters, determined by different infiltration patterns of TIICs, influence the prognosis of HCC, we therefore hypothesized that some of the cells were the key factors to prognosis, which was validated in the latter part.

Simultaneously, we also tried to explore the associations between immune checkpoint molecules expression and TMB among immune clusters. In this study, we found that PD-1, CTLA-4 and IFNG expression and TMB was higher in Cluster 1, and moderate in Cluster 2, lower in Cluster 3 and Cluster 4. We also found that CD8+ T cells and CD4+ memory activated T cells were strongly correlated with immune checkpoint molecules. Indeed, these two cells have important function in the antitumor immune response, including the antigen presentation by CD4+ memory activated T cells and cell killing by CD8+ T cells [4]. That means, Cluster1, even with a large amount of T cell, especially CD8+ T cells, infiltration in the tumor, high expression of immune checkpoint molecules would hinder their function, leading to an immunosuppressive and exhausted tumor microenvironment. Therefore, we would assume that application of immune checkpoint inhibitors would render a survival benefit for these HCC patients.

In the univariate Cox regression analysis, we found that macrophages M1 was significantly associated with improved outcome. Researches have shown in HCC that altering the polarization of TAM into M1 phenotype inhibits tumor growth [33, 34]. In addition, blockade of PD-1/PD-L1 in vivo increases macrophage phagocytosis, reduces tumor growth and prolong the survival of mice [35]. Tregs was increased in tumor tissue than in adjacent normal tissue, and associated with poorer outcome in our study. Previous studies have identified higher Tregs in chronic HBV, HCV infection and autoimmune hepatitis patients [36–38], emphasizing the vital role of Tregs as an immune suppressor in HCC development and a target for the prevention and treatment of HCC. Inhibition of Tregs-induced suppression was effective in rescuing anti‐tumor immune response in HCC and other kinds of cancers [8, 39–43]. Also, combination of PD-1/CTLA-4 blockade and depletion of Tregs in anti-tumor therapy have been shown to be effective [44, 45]. Our data confirmed and extended the findings from previous studies that M1 and Tregs were associated with clinical outcomes and provide potential therapeutic targets in HCC. Also, latest studies found that different modes of immune infiltration, such as NK cells, DCs and different immune microenvironment subtypes, were associated with clinical outcomes and immunotherapy response[46–48]. Further research is required to determine whether they could be new targets of immunotherapy against HCC. Additionally, we demonstrated that the Tregs was an independent prognostic factor of OS in HCC patients. Therefore, we combined Tregs and other clinical features (age, sex and TNM stage) to build a nomogram. The predicted probabilities of the nomogram were highly consistent with the actual probabilities for OS in HCC patients. It provides a complementary perspective on individual patients and might be a useful tool in clinical parctice.

Several limitations should be addressed in this study. In order to increase our sample size, we combined the transcriptomes from TCGA, ICGC and GEO datasets. Although statistical methods have been applied to eliminate cohort bias, these data are still heterogeneous in some level. Also, to obtain clinical outcome of all the studies, we inevitably lost some useful information, thus only used OS to analyze the outcome. In addition, genomic data of HCC patients received immunotherapy is still lacking for analysis, which can be a potential direction for future research.

In summary, our analysis has characterized the TIICs infiltration in HCC, identified the clusters by integrating the infiltration of 22 TIICs, which had prognostic significance. The nomogram based on Tregs could be used as a predictive tool to identify HCC patients with different survival.

## Supporting information

S1 Checklist STROBE statement—Checklist of items that should be included in reports of observational studies. (DOCX)

S1 Fig Proportions of tumor infiltrating immune cells between HCC and paired normal tissue. (TIF)

S2 Fig Selection of the number of clusters. (A) Elbow and Silhouette methods for each tested number of clusters; (B) Dendrogram of the clusters.
(TIF)

S3 Fig Distribution of each tumor infiltrating immune cell in immune clusters. (TIF)

S4 Fig Box plots summarizing HAVCR2, IL-2 and TGFB1 expression in different immune clusters. (TIF)

S5 Fig Kaplan-Meier curve of patients within different proportion of tumor-infiltration immune cells. The high and low groups were separated by the median value of each cell.
(TIF)

S1 Table Univariable Cox regression survival analysis in HCC patients. (DOCX)
