# Supplementary figures and images for: Tumor-infiltrating immune cells in hepatocellular carcinoma: Tregs is correlated with poor overall survival
Source: PLoS One. 2020 Apr 2;15(4):e0231003. doi: 10.1371/journal.pone.0231003 (PMC7117689; doi:10.1371/journal.pone.0231003)

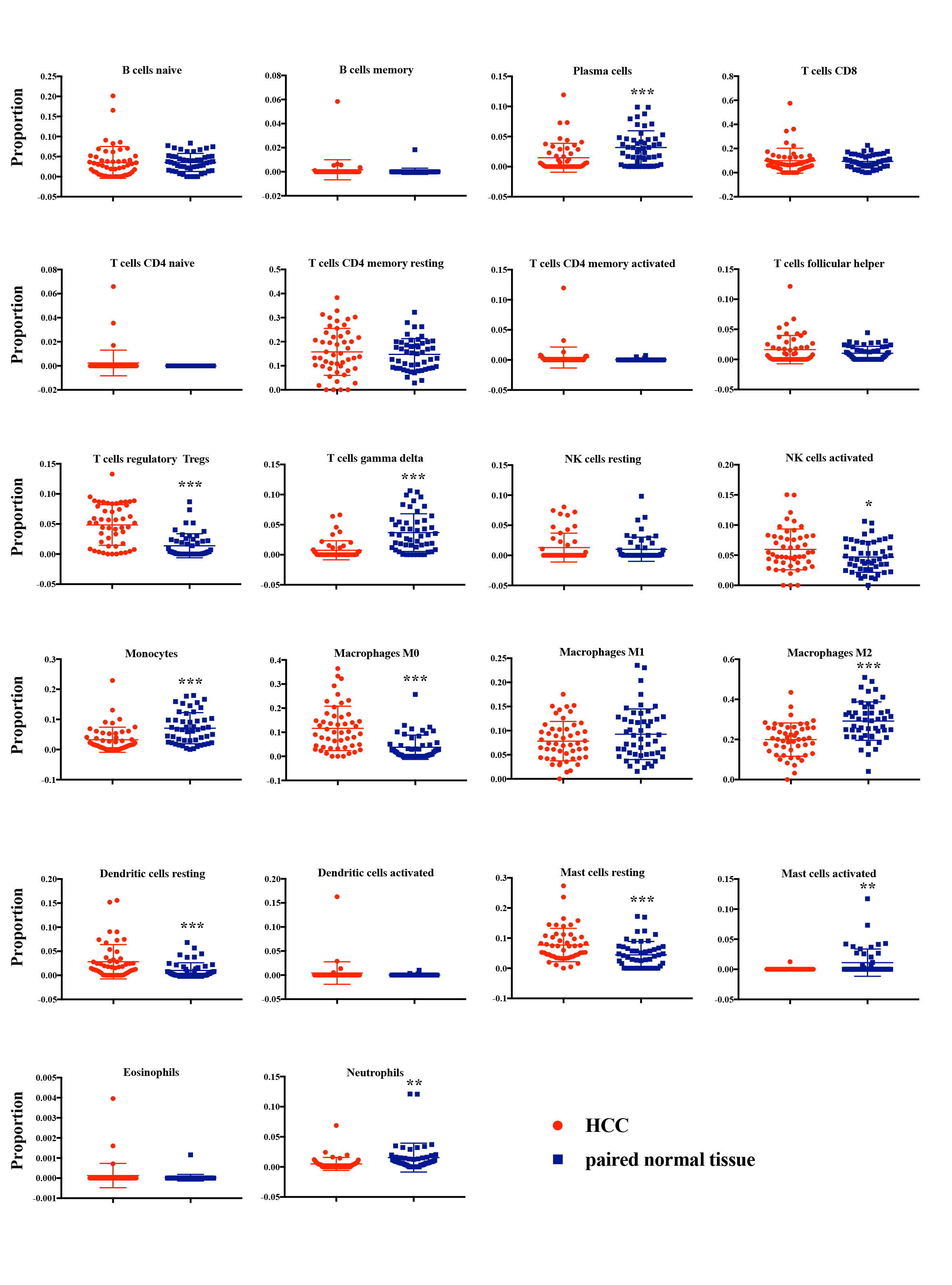

Supplement: S1 Fig — (TIF) [file pone.0231003.s002.tif]

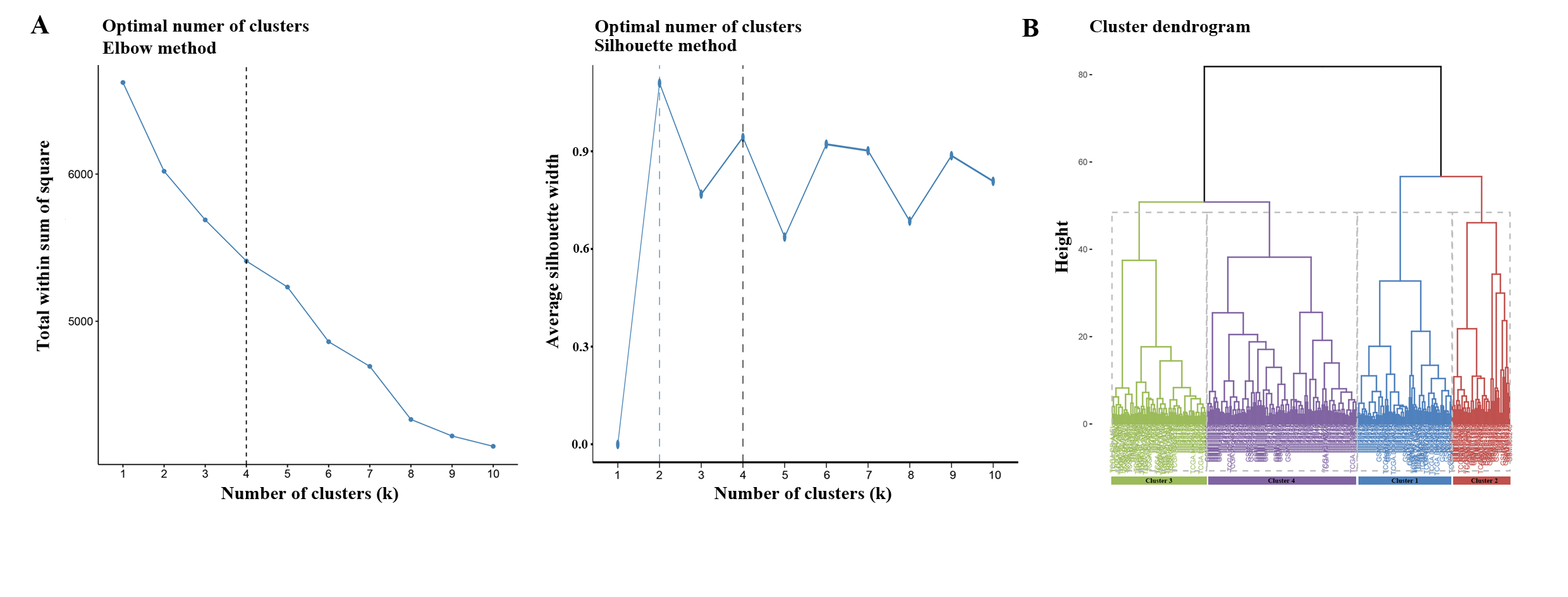

Supplement: S2 Fig — (A) Elbow and Silhouette methods for each tested number of clusters; (B) Dendrogram of the clusters. (TIF) [file pone.0231003.s003.tif]

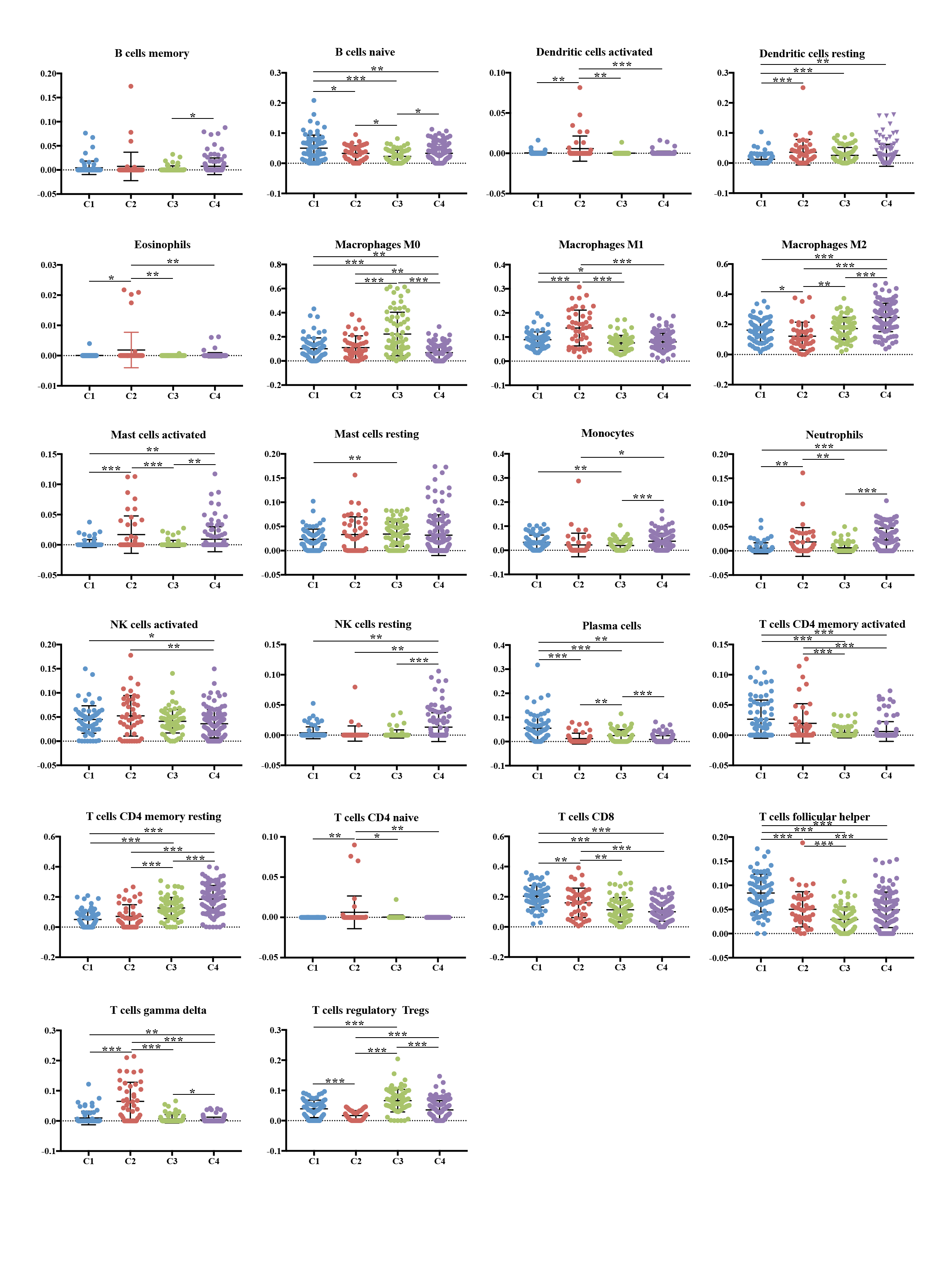

Supplement: S3 Fig — (TIF) [file pone.0231003.s004.tif]

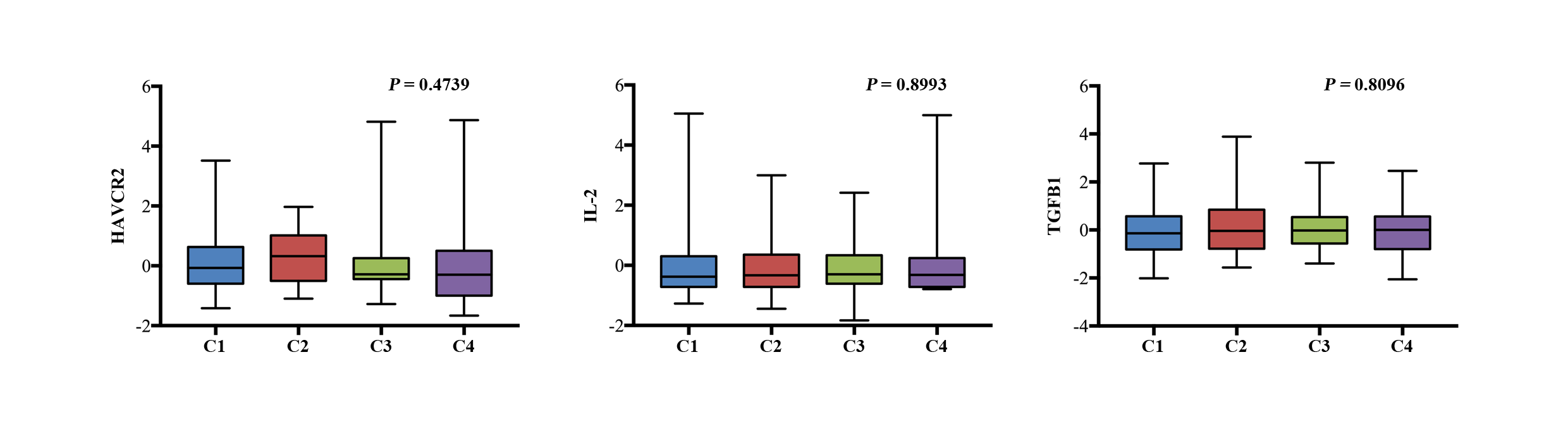

Supplement: S4 Fig — (TIF) [file pone.0231003.s005.tif]

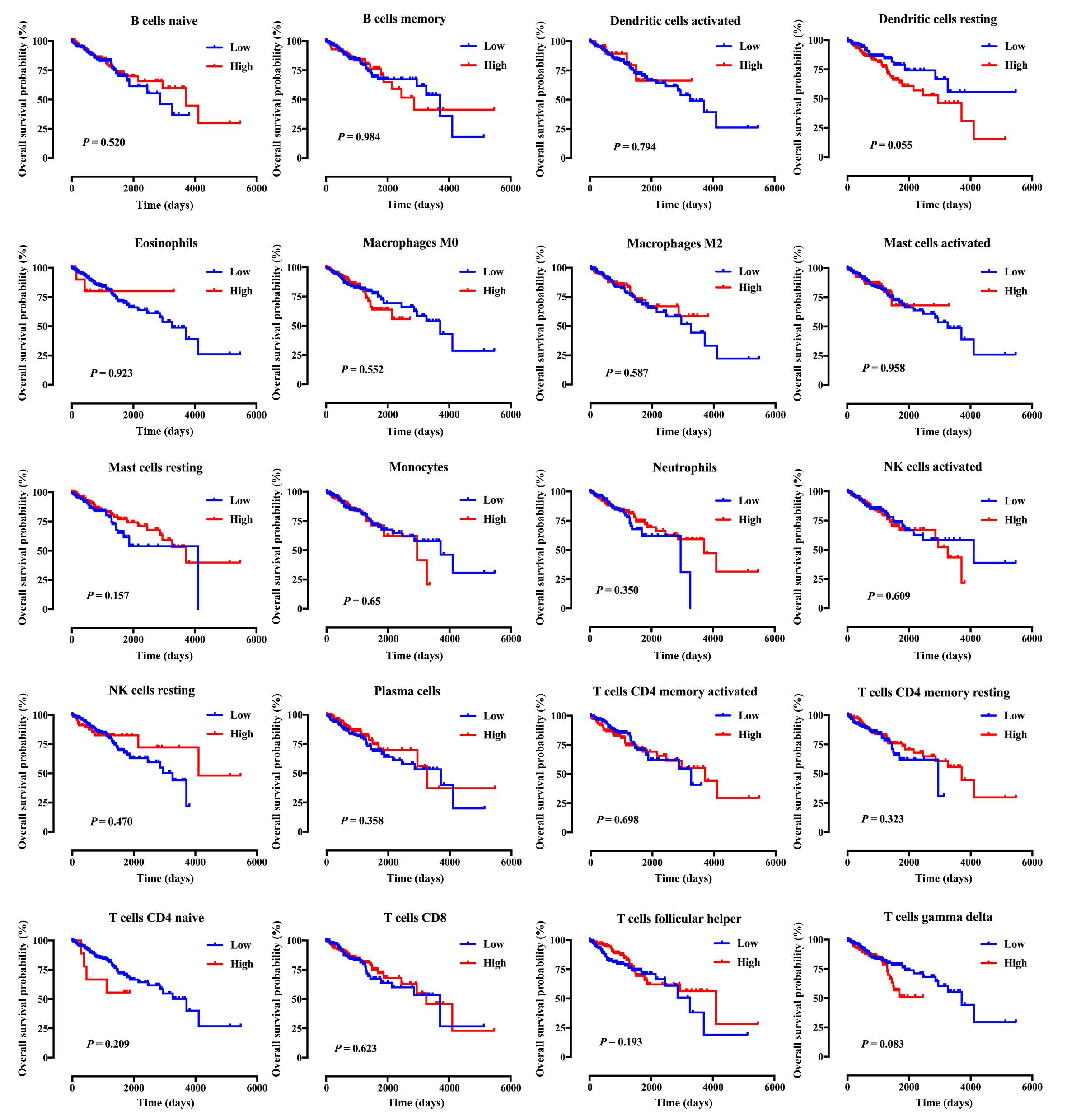

Supplement: S5 Fig — The high and low groups were separated by the median value of each cell. (TIF) [file pone.0231003.s006.tif]
