# Supplementary material for: Tumor-infiltrating immune cells in hepatocellular carcinoma: Tregs is correlated with poor overall survival
Source: PLoS One. 2020 Apr 2;15(4):e0231003. doi: 10.1371/journal.pone.0231003 (PMC7117689; doi:10.1371/journal.pone.0231003)
Supplement: S1 Table — (DOCX) [file pone.0231003.s007.docx]

| **Table S1.** Univariable Cox regression survival analysis in HCC patients. | | | | |
| --- | --- | --- | --- | --- |
| **Variable** | **Categories** | **Hazard ratio** | **95% CI** | ***P*-value** |
| PDCD1 | Continuous variable | 1.002 | 0.747-1.344 | 0.988 |
| CD274 | Continuous variable | 0.936 | 0.682-1.286 | 0.684 |
| CTLA4 | Continuous variable | 0.99 | 0.74-1.325 | 0.946 |
| HAVCR2 | Continuous variable | 0.925 | 0.669-1.279 | 0.636 |
| LAG3 | Continuous variable | 1.042 | 0.78-1.391 | 0.782 |
| IL2 | Continuous variable | 0.913 | 0.683-1.221 | 0.54 |
| IFNG | Continuous variable | 1.018 | 0.761-1.363 | 0.902 |
| TGFB1 | Continuous variable | 0.953 | 0.709-1.281 | 0.748 |
